# The Effect of Waste Ballast Aggregates on Mechanical and Durability Properties of Standard Concrete

**DOI:** 10.3390/ma16072665

**Published:** 2023-03-27

**Authors:** Hasan Erhan Yücel, Maciej Dutkiewicz, Fatih Yıldızhan

**Affiliations:** 1 Civil Engineering Department, Engineering Faculty, Niğde Ömer Halisdemir University, Niğde 51240, Turkey; 2 Faculty of Civil and Environmental Engineering and Architecture, Bydgoszcz University of Science and Technology, 85-796 Bydgoszcz, Poland; 3 Civil Engineering Department, Engineering Faculty, Gaziantep University, Gaziantep 27310, Turkey

**Keywords:** standard concrete, waste ballast, mechanical properties, durability properties

## Abstract

The acquisition and transportation of aggregate exacerbate the negative impact of concrete on the environment, and waste materials are considered an effective solution to this crucial problem. One of these waste materials is waste ballast (WB), which is needed for new infrastructure along with increasing rail track technology. In this study, the effect of WB aggregate (which is basalt-based) on the mechanical and durability properties of standard concrete was examined. Coarse aggregate was replaced with WB aggregate at the rates of 50%, 75% and 100%. The slump, compressive strength, flexural strength, capillary water absorption, rapid chloride permeability and water penetration tests on the mixtures were performed. According to the results of this study, the utilization of WB improved the compressive strength and flexural strength of the mixtures by about 15% and 7%, respectively. Moreover, the capillary water absorption, rapid chloride permeability and water penetration values of all the concrete mixtures with WB were lower than the control mixture. In addition, the correlation relations between the mechanical and durability properties indicated that they have a strong relationship with each other. All the results of this study demonstrated that the utilization of WB instead of coarse aggregate improved the mechanical and durability properties of concrete. WB can also provide a more sustainable material formation by minimizing the negative environmental effects of concrete production.

## 1. Introduction

Industrialization and related rapid urbanization have provided health, economic and technological development and also have led to many changes in human life [1,2]. One of these changes has been in the construction industry [3]. In the 19th and 20th centuries, the construction sector became one of the largest industries, with rapid developments such as concrete, steel-reinforced concrete, prestressed concrete, high-strength concrete and fibre-reinforced concrete [1,4]. This rapid development has made concrete the second most-used material after water [1]. Although the widespread use of concrete has provided advantages such as low cost, high compressive strength and durability, on-site or precast production, etc. [5], it has caused many negativities [6]. It is possible to deal with these negatives under two main headings: the excessive construction/demolition waste generated from aged structures and the environmental negative effects of concrete production.

Construction/demolition waste is increasing day by day because of the increase in the world’s population and the development of construction technology. For example, a total of 2533 million tons of waste was generated in the European Union, and about 922 tons of this waste belonged to construction/demolition waste in 2016 [7]. The amount of waste was 2.01 billion tons worldwide in 2016, and it is expected that the waste will increase to 3.40 billion tons by 2050 [8,9]. Furthermore, the landfill requirement for municipal solid waste disposal in India is expected to reach 169.6 km^2^ by 2047. This area is approximately eight times higher than in 1997 [2,10,11]. These wastes cause serious health, economic and environmental problems [8,12,13]. The recycling rate of construction/demolition waste in EU countries varies and the average recycling rate of construction/demolition waste in 27 EU countries is 55% [14,15]. This situation shows that the recycling rate of construction/demolition waste should be increased.

Another crucial problem is the negative environmental effects of concrete production. Cement production is responsible for 8% of the total CO_2_ emissions [1] and 36% of the CO_2_ emitted globally by construction activities [6,16]. The acquisition and transportation of aggregate further increases the negative effect of concrete [17] and concrete is responsible for 10% of the total CO_2_ emissions [18,19,20]. With the increasing demand, the need for these materials is constantly increasing. The annual concrete consumption is more than 10 billion tons [21,22,23], and it is expected to increase to about 18 million by 2050 [22,24]. The annual cement requirement for the concrete industry is 1.5 billion tons, the aggregate is 10–20 billion tons and the water requirement is approximately 1 million tons [21,25,26]. In addition, the global aggregate use in the construction industry reached 48 billion tons [27,28]. Most aggregates are obtained from natural deposits. For example, the rate of aggregate obtained from natural deposits is 91% in Europe [29]. The acquisition of natural aggregate causes erosion of the river deltas and coastlines [21,30]. Moreover, a shortage of aggregate is another problem to be considered [31]. Therefore, the International Energy Agency recommends the use of different materials instead of natural aggregates [21,32]. Considering all these problems, it can be said that the best way is to recycle waste and use it instead of aggregate [33].

The idea of more reliable solutions and materials that meet the principle of sustainable development is important for the development of new concretes and mortars [34,35].

The use of waste materials instead of aggregate has been tried in many studies [36,37,38,39]. Moreover, many countries have started the application of utilizing recycled aggregate in this sense. For example, the first attempts to recycle construction/demolition waste started in Germany in the 1980s. Later, many countries made progress toward this aim [36]. One of the construction/demolition wastes used in these applications is waste ballast (WB). A large amount of WB is formed due to the increasing need for new infrastructure along with developing rail track technology [40]. For example, Indian railways supplied around eight million cubic meters of ballast in 2017–2018 [41,42]. Ballast materials are high quality igneous or metamorphic rocks. In addition, their particle size, particle shape, strength, hardness and resistance properties are of a quality that satisfies certain criteria [43]. Generally, basalt and granite are used as ballast materials [44]. Korkanç and Tuğrul [45] also stated that the mechanical properties of basalts are appropriate for use as aggregates for concrete production. Therefore, positive results were obtained in the applications of recycling WB. WB was tested for self-compacting recycled aggregate concrete [7], recycled aggregate mortar [46], over-burnt brick ballast aggregate concrete [3] and preplaced aggregate concrete [47]. Furthermore, it was tested for the partial replacement of coarse aggregate in concrete to some extent (up to 20%) [17].

As of 2020, there was a total of 12,803 km of national railway network in Turkey, of which there were 11,590 km of conventional lines and 1213 km of high-speed lines [48]. Considering Turkey in general, it is thought that approximately 5440 tons of waste ballast is produced annually. For this reason, the recycling of waste ballast, which is produced in large quantities in Turkey, is very important. In addition, since it is known that high quality aggregates such as basalt are generally used as a ballast aggregate in Turkey, it was thought that the ballast aggregate obtained as waste could be used in concrete production.

The capability of using the higher amount of WB and its evaluation based on its strength and durability properties in conventional concrete is a promising issue. The high available amount, the superior quality, the targeting of high rates in recycling and the positive results obtained from WB in the literature support this idea. In the literature, there are very limited studies that have observed WB aggregates used in concrete, and in these studies, the ratio of replacement with normal aggregates is at a maximum 40%. In addition, these studies only focused on the strength, freezing–thawing and shrinkage properties. Within the scope of this study, the effect of WB aggregate (which is basalt-based) on the mechanical and permeability properties, which are very important for the durability properties of concrete, was investigated. Coarse aggregate was replaced with WB aggregate at the rates of 50%, 75% and 100%. In addition, C30/37 concrete was produced as the control mixture. The slump, compressive strength, flexural strength, capillary water absorption, rapid chloride permeability and water penetration tests on the mixtures were performed and the effect of WB was evaluated. Finally, correlation relations between mechanical and durability properties were determined.

## 2. Experimental Program

### 2.1. Materials

**Figure 1:**
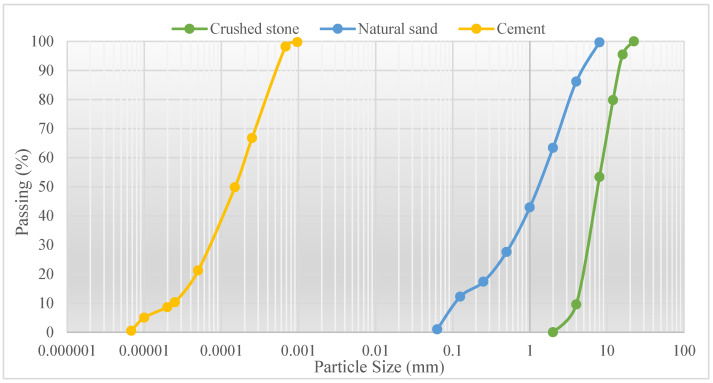
Particle size distribution of aggregate.

**Figure 2:**
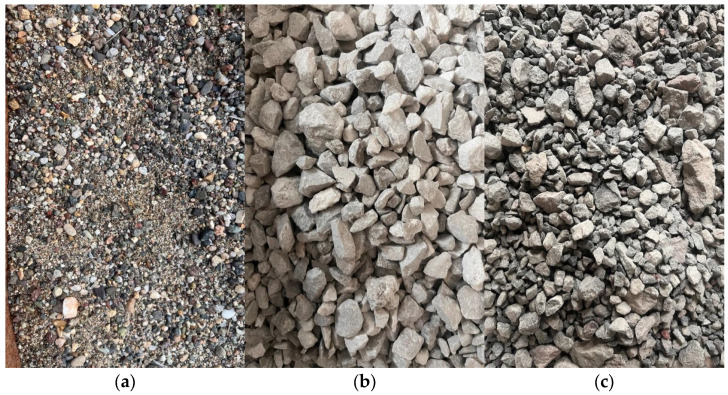
(**a**) Natural sand, (**b**) Crushed stone and (**c**) WB.

Standard CEM I 42.5 R Portland cement was used in all mixtures. The chemical composition and physical properties of Portland cement are presented in Table 1. Figure 1 shows the particle size distribution of aggregate and cement. Figure 2 presents natural sand, crushed stone and WB. Natural sand aggregate between 0–4 mm was used as a fine aggregate. The specific gravity of natural sand is 2.67, its water absorption rate is 2.65% and its natural humidity is 0.8%. Crushed stone aggregate between 4–16 mm was used as coarse aggregate. The specific gravity of crushed stone is 2.65, its water absorption rate is 0.9% and its natural humidity is 0.81%. Finally, the specific gravity of the WB aggregate is 2.67 and its water absorption rate is 0.7%. As can be seen in Figure 2, WB has an irregular and elongated shape.

In order for an aggregate to be used as a ballast, the mass losses obtained in the Los Angeles fragmentation resistance determination, frost resistance determination and abrasion resistance determination tests must be equal to or lower than 14%, 3% and 12%, respectively [49]. The above-mentioned tests were applied to the aggregates used as ballasts at certain periods, and the aggregates below these values were stored as waste ballasts. The waste ballast aggregate stored in the Kayseri region in Turkey was used as the WB in this study. The WB was obtained as a result of the wear of the basalt aggregate produced in the Kayseri region as a result of using it as a ballast.

### 2.2. Mix Design of Concrete

Four mixtures were prepared with the proportions shown in Table 2. When the studies in the literature were examined, it was seen that the ballast aggregate was replaced with normal aggregate at the maximum rate of 40% in the concrete mixtures [3,50]. This rate was 25%, 50%, 75% and 100% in the studies where other recycled aggregates were used, especially for basalt-based recycled aggregates [7,18,29,46,51,52,53]. Because of this, the aggregate used in this study was high-quality waste basalt-based ballast aggregate, and the coarse aggregate was replaced with WB at a rate of 50%, 75% and 100%, respectively. WB50 refers to 50%, WB75 refers to 75%, and WB100 refers to 100% utilization of WB. For the aggregate, the most ideal mixture was found with the use of 60% crushed stone aggregate and 40% natural sand. After determining the aggregate ratio to be used, the targeted concrete compressive strength of the concrete to be produced was determined as C30/37. Firstly, the slump tests on the mixtures were performed according to ASTM C143. The slump value was used to check whether the design was performed correctly or not, because it is a significant parameter in the design of a concrete mixture. The experiment was performed as follows: fresh concrete was placed in the mold in three layers, with each layer filling approximately one-third of the mold. Each placed layer was tamped 25 times with a tamping rod. Mold was slowly pulled vertically upwards and the slump values of the mixtures were measured.

### 2.3. Test Procedures

#### 2.3.1. Compressive and Flexural Strength Test

**Figure 3:**
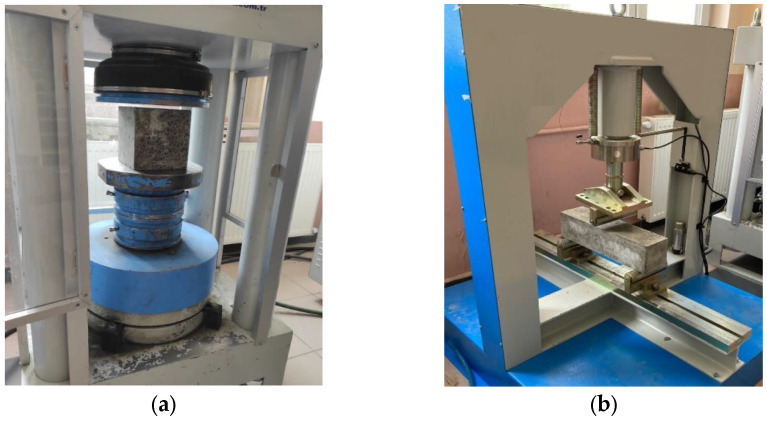

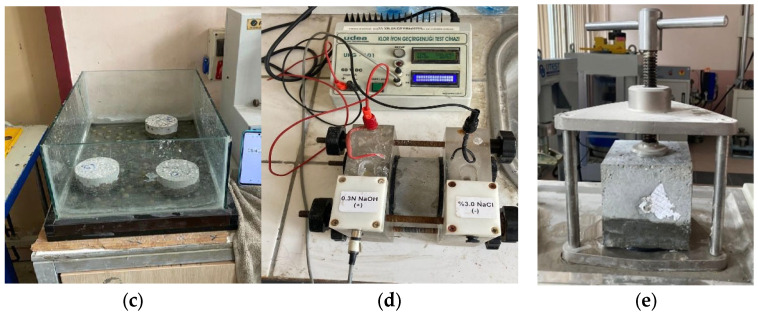
(**a**) Compressive, (**b**) flexural, (**c**) capillary water absorption, (**d**) rapid chloride permeability and (**e**) water penetration test setups.

The compressive and flexural strengths of the mixtures were determined with respect to ASTM C 39 [54] and ASTM C 78 [55] at 7 and 28 days, respectively. The compressive and flexural test setups are displayed in Figure 3a,b, respectively. Six cubic specimens with the dimension of 150 mm × 150 mm × 150 mm were tested to determine the compressive strength of each mixture. For the flexural test, six prism specimens with the dimension of 100 mm × 100 mm × 500 mm were used. The compressive and flexural strengths were determined considering the average values of these specimens.

#### 2.3.2. Capillary Water Absorption

The capillary water absorption of the mixtures was determined with respect to ASTM C1585 [56] at 7 and 28 days. The capillary water absorption test procedure is shown in Figure 3c. Three samples of each mixture were kept in a 100 °C oven for 24 h and completely dried. Then, the samples were coated with epoxy to prevent water absorption from their surfaces, except for the surface touching the water. Finally, the sides of the samples that were not coated with epoxy were placed on the tray in contact with water and their weights were measured at regular periods.

#### 2.3.3. Rapid Chloride Permeability

The rapid chloride permeability tests of the specimens were carried out according to ASTM C1202 [57] at 7 and 28 days. The rapid chloride permeability test setup is shown in Figure 3d. The tests were performed on three specimens with a height of 50 mm and a diameter of 100 mm. The specimens were placed between two cells, one with a 3.0% salt (NaCl) solution and the other with a 0.3 M sodium hydroxide (NaOH) solution. Finally, the electrodes were immersed in the cells with a voltage of 60 V and the rapid chloride permeability was calculated according to the charge that passed through the specimens.

#### 2.3.4. Water Penetration Test

The depth of water penetration was determined with respect to BS EN 12390-8 [58]. The water penetration test procedure is displayed in Figure 3e. This experiment was carried out on standard cured samples at 7 days and 28 days, respectively. The samples removed from curing were allowed to dry for approximately 5 h in the laboratory environment. Silicone material was used for the side surface of the samples. The samples, which were coated with silicone material, were left to dry. In the experiment, water was applied to the bottom surface of the samples under 5 bar pressure for 72 h. Afterwards, the samples were removed from the device and the depth of the water penetration in the hardened concrete specimens under pressure was determined by splitting the sample in half, perpendicular to the surface to which the pressurized water was applied.

## 3. Results and Discussion

### 3.1. Compressive Strength Test

**Figure 4:**
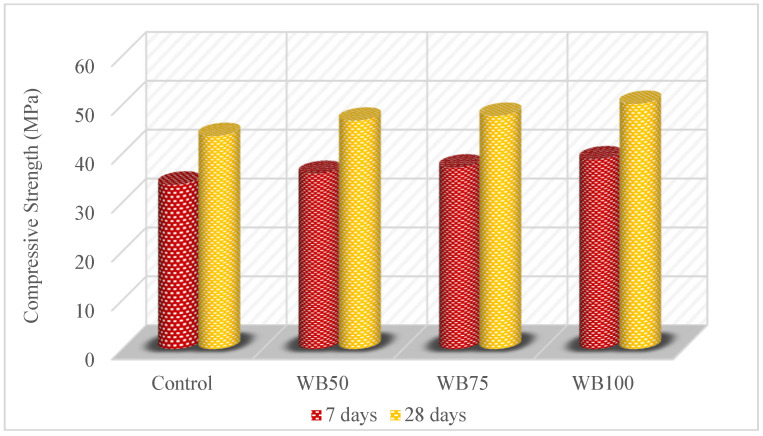
Compressive strength of mixtures.

The compressive strength test results of the mixtures are shown in Figure 4. As the amount of WB increased, the compressive strength of the mixtures increased. The compressive strength increased by 6.57%, 10.41% and 15.44% for WB50, WB75 and WB100, compared to the control mixture at 7 days, respectively. Moreover, at 28 days, the compressive strength increased in the range of 7.23%, 9.45% and 15.08%, respectively. According to these results, the WB positively affected the compressive strength. This improvement can be attributed to the good quality of the WB [7,59] and also to the irregular particle shape of the WB, which causes an increment in the adhesion between the paste and WB [51]. There are some studies in the literature about the utilization of WB or basalt that increases the compressive strength. Dasari et al. [17] used 5%, 10%, 15% and 20% WB instead of coarse aggregate. The compressive strength increased as the amount of WB increased. Aja et al. [7] indicated that concrete with recycled ballast could fulfil the requirement for the compressive strength of a slab track. Özturan and Çeçen [60] stated that concrete with basalt aggregate provides a higher strength than concrete using limestone and gravel aggregate. Kishore et al. [61] tested the basalt replacement of coarse aggregate at a rate of 0% to 100% by steps of 25% increments and found that the utilization of basalt increased the compressive strength. Boğa and Şenol [52] used basalt instead of crushed stone for high-strength self-compacting concrete. The basalt replacement of crushed stone at rates of 25%, 50%, 75% and 100% were tested, and the 100% utilization of basalt had the highest compressive strength, and this was also higher than the control mixture. Ajdukiewicz and Kliszczewicz [62] also supported these results and reported that the concrete with waste basalt and natural basalt had similar mechanical properties. These findings showed that waste basalt, which is a type of ballast material and natural basalt, gave similar results and also improved the compressive strength. According to the test results, the WB used in the study increased the compressive strength, although it was less than that reported by studies in the literature. This can be explained by the use of WB at a much higher replacement ratio compared to the literature.

### 3.2. Flexural Strength Test

**Figure 5:**
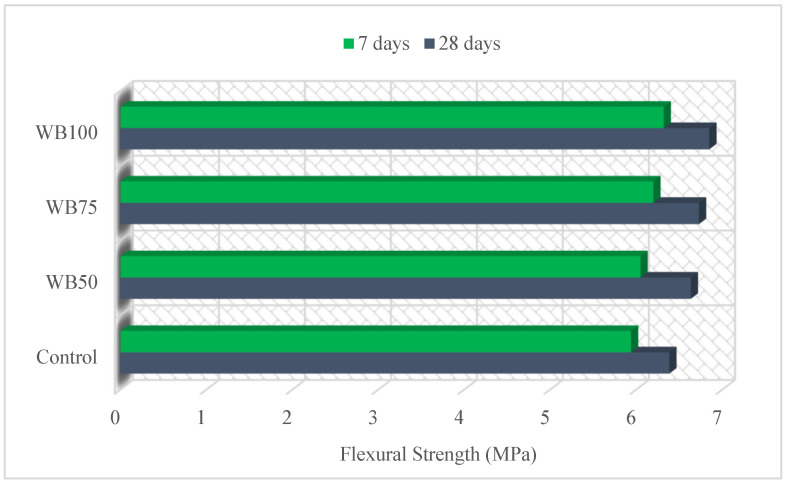
Flexural strength of mixtures.

The flexural strength test results of the mixtures are given in Figure 5. The minimum and maximum flexural strengths were obtained from the control mixture and WB100 at 7 and 28 days, respectively. The flexural strength of WB100 at 7 and 28 days, increased in the ratios of 6.40% and 7.20% compared to the control mixture, respectively. WB enhanced the flexural strength of the mixtures. This improvement can be explained by the irregular and elongated shape of WB [51]. Similarly, Dasari et al. [17] studied a 20% WB replacement of coarse aggregate and an increment of 17.49% and 19.11% was observed in the 7- and 28-day flexural strengths, respectively. Ali et al. [3] tried over-burnt brick ballast instead of coarse aggregate and the utilization of 20% WB increased the 28-day flexural strength by 30% compared to the control mixture. Özturan and Çeçen [60] expressed that concrete with basalt aggregate provides a higher strength than concrete using gravel aggregate. According to the test results, the WB used in the study increased the flexural strength, although it was less than that reported by studies in the literature. This can be explained by the use of WB at a much higher replacement ratio compared to the literature.

### 3.3. Capillary Water Absorption

**Figure 6:**
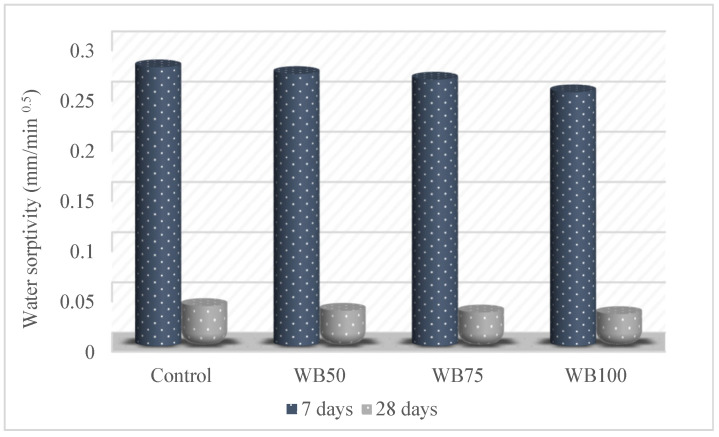
Capillary water absorption of mixtures.

The capillary water absorption values of the mixtures are graphically presented in Figure 6. As can be seen in Figure 6, the capillary water absorption value decreased when the amount of WB increased. The 7-day and 28-day capillary water absorption values of the control mixture were 0.278 mm/min^0.5^ and 0.039 mm/min^0.5^, respectively, whereas the 7-day and 28-day capillary water absorption values of the WB100 were 0.253 mm/min^0.5^ and 0.032 mm/min^0.5^, respectively. Therefore, it was determined that there was a 9% and 18% reduction in the capillary water absorption values for 7 and 28 days, respectively. This finding shows that WB reduced the capillary water absorption and composed a more impermeable concrete than the control mixture. This situation can be explained by the low void and water absorption values of WB [63,64]. When the test results were examined, it was determined that the WB used in this study had a more positive effect on the capillary water absorption than the studies in the literature.

### 3.4. Rapid Chloride Permeability

**Figure 7:**
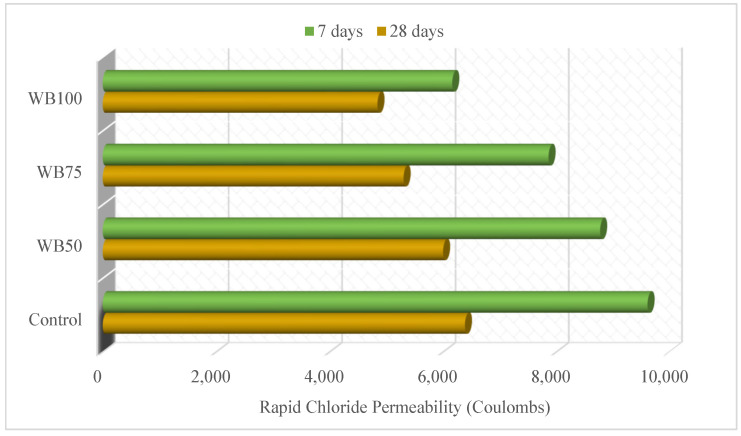
Rapid chloride permeability of mixtures.

The rapid chloride permeability test results of the mixtures are plotted in Figure 7. The 7-day and 28-day rapid chloride permeability values were in the range of 6161–9613 and 4842–6384 coulombs, respectively. The rapid chloride permeability value of WB100 was 35.91% less at 7 days and 24.10% at 28 days, compared to the control mixture. The rapid chloride permeability decreased as the WB increased. This finding indicates that the WB improved the impermeability of the concrete and formed a more durable concrete. This improvement was attributed to the formation of a high-density WB mixture due to WB [51]. Korkanç and Tuğrul [45] stated that the durability properties of basalts can fulfil the requirement for use as an aggregate for concrete production.

### 3.5. Water Penetration Test

**Figure 8:**
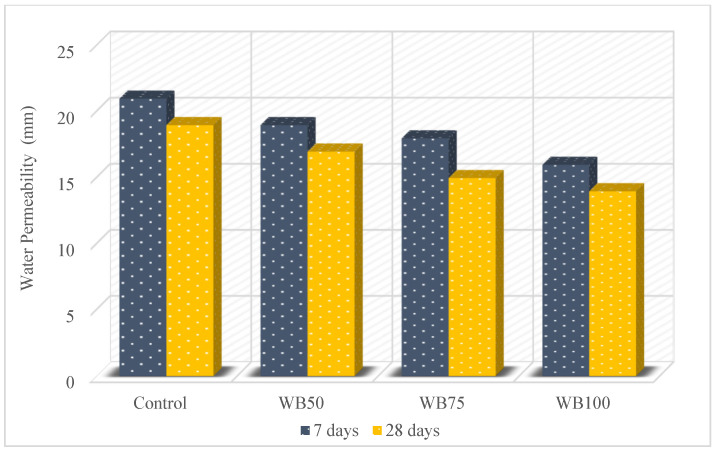
Water penetration of mixtures.

The water penetration values of the mixtures are given in Figure 8. The maximum 7-day water penetration result was obtained as 21 mm for the control mixture and the minimum was 16 mm for WB100, while the maximum was the 28-day water penetration result obtained as 19 mm for the control mixture and the minimum was 14 mm for the WB100. Therefore, it was determined that there was a 23.8% and 26.3% reduction in the water penetration values for 7 and 28 days, respectively. All mixtures were more impermeable than the control mixture. This condition can be clarified by noting that the density of the WB is higher than the crushed stone [51]. This situation can be also observed in the literature studies. Aja et al. [7] stated that the penetration of concrete with WB satisfied the standards. When the test results were examined, it was determined that the WB used in this study caused a greater decrease in the water penetration value than those of the studies in the literature.

### 3.6. Relationships between the Properties of Concrete Mixture with WB

**Figure 9:**
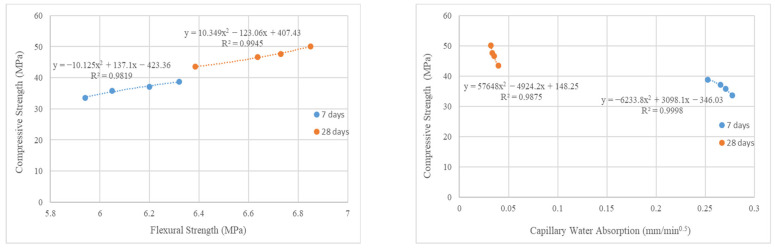

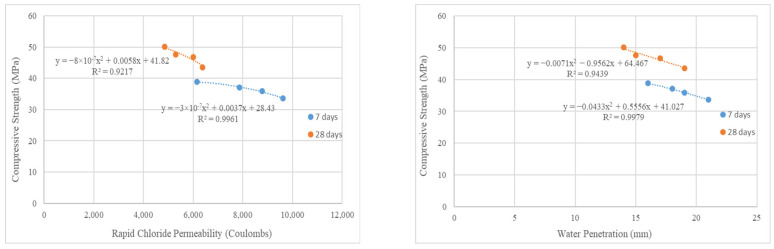
Correlation relations between mechanical and durability properties with compressive strength.

**Figure 10:**
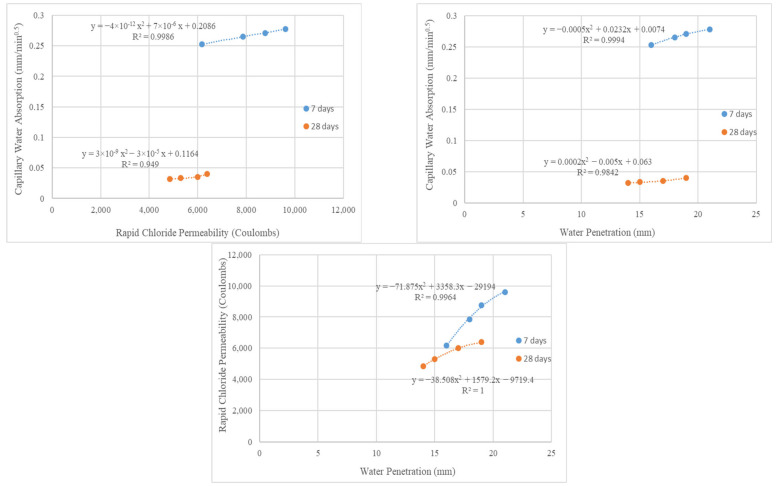
Correlation relations between durability properties.

The correlation (relationship) of test results is one of the methods frequently used in studies to evaluate experimental data [65]. For this study, the correlation relations between the mechanical and durability properties with the compressive strength at 7 and 28 days are given in Figure 9. In addition, the correlation coefficients (R^2^) between the mechanical and durability properties with the compressive strength are given in Table 3. The analysis results show that there was a high correlation between the mechanical and durability properties with the compressive strength. Because of this, the R^2^ was determined as greater than 0.90 for all the relations. All the correlation relations were determined as polynomial. Furthermore, the correlation relations between the durability properties at 7 and 28 days are shown in Figure 10. Furthermore, the correlation coefficients (R^2^) between the durability properties are given in Table 4. The correlation coefficients pointed out that the durability properties of theconcrete with WB had strong relationships (R^2^ > 0.95) with each other.

## 4. Conclusions

In this study, the effect of using WB instead of coarse aggregate on the mechanical and durability properties of standard concrete was investigated and the following results were obtained:

- When the mechanical properties of the mixtures were considered, the utilization of WB improved the compressive strength of the mixtures. This improvement was approximately 7%, 10% and 15% for WB50, WB75 and WB100 compared to the control mixture, respectively. This increment can be attributed to the high quality and irregular particle shape of WB, and the increase in adhesion between the paste and WB.
- According to the flexural strength test results, WB enhanced the flexural strength of the mixtures. The flexural strength of WB100 was greater than that of the control mixture in the ratios of 6.40% and 7.20% at 7 and 28 days, respectively. This improvement can be explained by the irregular and elongated shape of WB.
- When the durability properties of the mixtures were taken into account, the capillary water absorption value reduced depending on the amount of WB. The capillary water absorption values of the control and WB100 were 0.278–0.039 mm/min^0.5^ and 0.253–0.032 mm/min^0.5^ for 7 and 28 days, respectively. This result indicates that WB influenced the impermeability of the concrete positively. This situation can be explained by the low void and water absorption values of WB.
- The rapid chloride permeability test value decreased as the WB increased. The rapid chloride permeability value of WB100 was 35.91% less at 7 days and 24.10% at 28 days, compared to the control mixture. This result shows that WB enhanced the durability properties. This improvement was attributed to the formation of a high-density concrete mixture with WB due to the WB.
- The water penetration results of the control mixture and WB100 at 7 days were 21 mm and 16 mm, respectively, while the water penetration results of the control mixture and WB100 at 28 days were 19 mm and 14 mm, respectively. These findings show that the concrete mixture with WB was more durable than the control mixture. This condition can be clarified by the fact that the density of the WB is higher than the crushed stone.
- When the correlation relations were considered, the R2 was determined as higher than 0.90 for the correlation between the mechanical and durability properties with the compressive strength. The correlation relations between the durability properties also had strong relationships with each other (R2 > 0.95).

All the results of this study demonstrated that the utilization of WB instead of coarse aggregate improved the mechanical and durability properties of standard concrete. This utilization (up to 100%) can not only enhance the properties of the concrete, but can also provide more sustainable concrete production.

## Figures and Tables

**Table 1:** Chemical composition and physical properties of Portland cement.

Chemical Composition (%)	Portland Cement
CaO	62.19
SiO_2_	19.83
Al_2_O_3_	5.31
Fe_2_O_3_	3.17
MgO	1.89
SO_3_	3.27
K_2_O	1.01
Na_2_O	0.21
Physical properties	
Loss of Ignition	2.98
Specific Gravity	3.15
Fineness (Blaine) (m^2^/kg)	326

**Table 2:** Mix design of concrete (kg/m^3^).

Materials	Control	WB50	WB75	WB100
Cement	486.96	486.96	486.96	486.96
Coarse Aggregate	955.41	477.71	238.85	-
Fine Aggregate	625.72	625.72	625.72	625.72
WB Aggregate	-	477.71	716.56	955.41
Water	236.66	236.66	236.66	236.66
Slump Value (mm)	180	178	177	177

**Table 3:** Correlation coefficients between mechanical and durability properties with compressive strength.

Properties	Compressive Strength	
	(R^2^) 7 Days	(R^2^) 28 Days
Flexural Strength	0.9819	0.9945
Capillary Water Absorption	0.9998	0.9875
Rapid Chloride Permeability	0.9961	0.9217
Water Penetration	0.9979	0.9439

**Table 4:** Correlation coefficients between durability properties.

Properties	Capillary Water Absorption		Rapid Chloride Permeability		Water Penetration	
	(R^2^) 7 Days	(R^2^) 7 Days	(R^2^) 7 Days	(R^2^) 28 Days	(R^2^) 28 Days	(R^2^) 28 Days
Capillary Water Absorption	1	1	0.9986	0.949	0.9994	0.9842
Rapid Chloride Permeability	0.9986	0.949	1	1	0.9964	1
Water Penetration	0.9994	0.9842	0.9964	1	1	1

## Data Availability

Not applicable.
